# Druggable Lipid Binding Sites in Pentameric Ligand-Gated Ion Channels and Transient Receptor Potential Channels

**DOI:** 10.3389/fphys.2021.798102

**Published:** 2022-01-04

**Authors:** Wayland W. L. Cheng, Mark J. Arcario, John T. Petroff

**Affiliations:** Department of Anesthesiology, Washington University in St. Louis, St. Louis, MO, United States

**Keywords:** lipid binding sites, pentameric ligand-gated ion channel, transient receptor potential channel, allosteric modulation, cryo-EM, photoaffinity labeling, native mass spectrometry, molecular dynamics simulations

## Abstract

Lipids modulate the function of many ion channels, possibly through direct lipid-protein interactions. The recent outpouring of ion channel structures by cryo-EM has revealed many lipid binding sites. Whether these sites mediate lipid modulation of ion channel function is not firmly established in most cases. However, it is intriguing that many of these lipid binding sites are also known sites for other allosteric modulators or drugs, supporting the notion that lipids act as endogenous allosteric modulators through these sites. Here, we review such lipid-drug binding sites, focusing on pentameric ligand-gated ion channels and transient receptor potential channels. Notable examples include sites for phospholipids and sterols that are shared by anesthetics and vanilloids. We discuss some implications of lipid binding at these sites including the possibility that lipids can alter drug potency or that understanding protein-lipid interactions can guide drug design. Structures are only the first step toward understanding the mechanism of lipid modulation at these sites. Looking forward, we identify knowledge gaps in the field and approaches to address them. These include defining the effects of lipids on channel function in reconstituted systems using asymmetric membranes and measuring lipid binding affinities at specific sites using native mass spectrometry, fluorescence binding assays, and computational approaches.

## Introduction

Lipids regulate the structure and function of ion channels, which are embedded in complex and dynamic lipid membrane environments. Classic examples include cholesterol activation of the nicotinic acetylcholine receptor (nAchR; Ochoa et al., 1983; Criado et al., 1984) and phosphatidylinositol activation of inward rectifying potassium (Kir) channels (Hilgemann and Ball, 1996; Shyng and Nichols, 1998). It is now known that all types of ion channels, from voltage-gated and transient receptor potential (TRP) channels to mechanosensitive channels, are regulated by lipids (Rosenhouse-Dantsker et al., 2012; Thompson and Baenziger, 2020). While the physiologic and pathophysiologic implications of these effects are not known in most cases, pharmacologically targeting mechanisms of lipid regulation, such as lipid binding sites, may provide a useful approach to treat channelopathies or other diseases (Reddy and Estes, 2016; Arul Prakash and Kamlekar, 2021; Payandeh and Volgraf, 2021).

Lipids may regulate ion channels by direct binding to specific sites or indirect effects on membrane physical properties (Cordero-Morales and Vasquez, 2018). The recent outpouring of ion channel structures by cryo-electron microscopy (cryo-EM) has revealed many structures with bound lipids. While these structures have provided striking insight into lipid-channel interactions, in few cases is it known whether lipid occupancy of these sites is functionally important. Interestingly, some of these sites are also known sites of action for other allosteric modulators or drugs, supporting the hypothesis that lipids modulate channel function by binding to these sites. For example, lipid densities have been observed in anesthetic binding sites in pentameric ligand-gated ion channels (pLGICs) and in the vanilloid binding site in transient receptor potential (TRP) channels. Here, we will review such lipid-drug binding sites from recent structural studies with a focus on pLGICs and TRP channels. We discuss potential implications of these shared lipid-drug sites for understanding drug action and structure-based drug design. As the structural data raise questions regarding the selectivity and functional significance of lipid binding to these sites, we highlight several approaches to address these questions in future research efforts.

## Lipid-Drug Binding Sites in Plgics

pLGICs, such as the nicotinic acetylcholine receptor (nAchR) and GABA(A) receptor (GABA_A_R), mediate synaptic neurotransmission and determine neuronal excitability. Over four decades ago, the nAchR from *Torpedo* muscle was the first ion channel found to be sensitive to its lipid environment. The nAchR requires cholesterol, phosphatidylethanolamine (PE), and anionic phospholipids, such as phosphatidylserine (PS), for maximal channel responses to agonist (Dalziel et al., 1980; Criado et al., 1982, 1984; Ochoa et al., 1983; Fong and McNamee, 1986; Hamouda et al., 2006). These lipid constituents specifically interact with the transmembrane domain (TMD) at putative annular and non-annular sites (Marsh and Barrantes, 1978; Marsh et al., 1981; Gonzalez-Ros et al., 1982; Jones and McNamee, 1988). The GABA_A_R is also modulated by cholesterol (Sooksawate and Simmonds, 2001a), certain phospholipids, such as phosphatidylserine (Hammond and Martin, 1987; Dostalova et al., 2010, 2014), as well as polyunsaturated fatty acids (Hamano et al., 1996; Nabekura et al., 1998), and neurosteroids (Belelli and Lambert, 2005). In contrast, the lipid sensitivities of other pLGICs, such as the serotonin receptor (5-HT3aR; Fan, 1995; Nothdurfter et al., 2013), glycine receptor (GlyR; Yang et al., 2008), and non-mammalian pLGICs (e.g., ELIC: Tong et al., 2019 and GLIC: Velisetty and Chakrapani, 2012) are less well-characterized. Nevertheless, pLGICs are undoubtedly lipid-sensitive channels. There now exists a large inventory of high-resolution structures encompassing all types of pLGICs, many of which show lipid-drug binding sites. Such sites provide a structural framework to begin clarifying mechanisms of lipid modulation in this family of ion channels.

**Figure 1:**
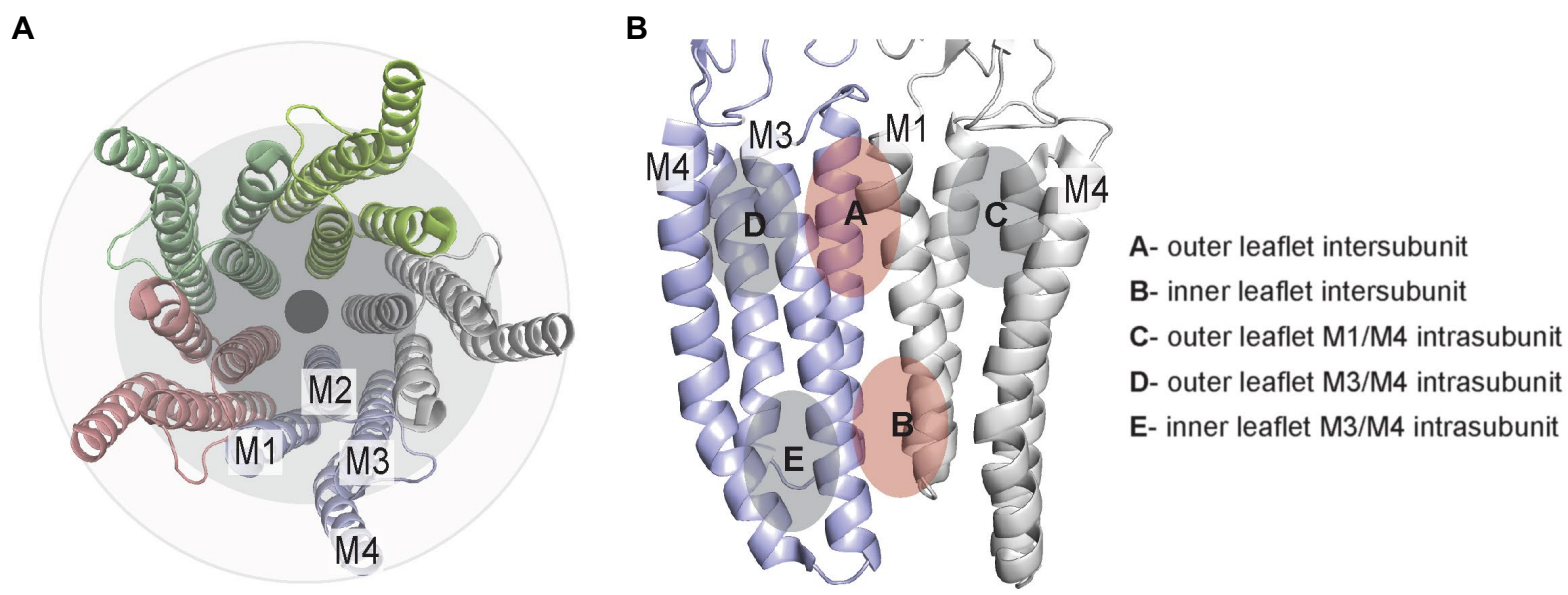
TMD lipid-drug binding sites in pLGICs illustrated using the GLIC structure (pdb 4hfi). **(A)** Top/extracellular view of the TMD helices showing the 3-ring organization. **(B)** Two adjacent TMD subunits illustrating the general location of inter- and intrasubunit sites in the outer and inner leaflet.

pLGICs consist of homo- or hetero-pentamers with four transmembrane helices per subunit. Orthogonal to the membrane, these helices are approximately organized as three rings, from the pore to the surrounding lipid membrane. The inner ring consists of transmembrane helix 2 (M2), which lines the channel pore, the intermediate ring consists of M1 and M3, and the outer ring consists of M4, which has the greatest exposure to lipids (Barrantes and Fantini, 2016; Figure 1A). Between these helices are grooves that are generally conserved among all pLGICs; M1 and M3 form an intersubunit groove, while M1 and M4 or M3 and M4 form intrasubunit grooves. These grooves are known binding sites for many allosteric modulators. In this review, we organize these sites as intersubunit and intrasubunit sites in the outer or inner leaflet (Figure 1B). This delineation (i.e., outer versus inner) is useful for discussion of these sites, although it may be an oversimplification for some deeply bound lipids, which may not clearly associate with lipids from one membrane leaflet. In addition, most pLGICs including the GABA_A_R and nAchR assemble as heteropentamers, increasing the number of unique sites within the pentameric assembly. For example, the most prevalent composition of synaptic GABA_A_R consists of two α1 subunits, two β2 or β3 subunits and one γ2 subunit with an arrangement of α1-β2-α1-β2-γ2. Thus, there are three distinct subunits and four distinct interfaces in the heteropentameric GABA_A_R.

### Outer Leaflet Intersubunit Site

**Figure 2:**
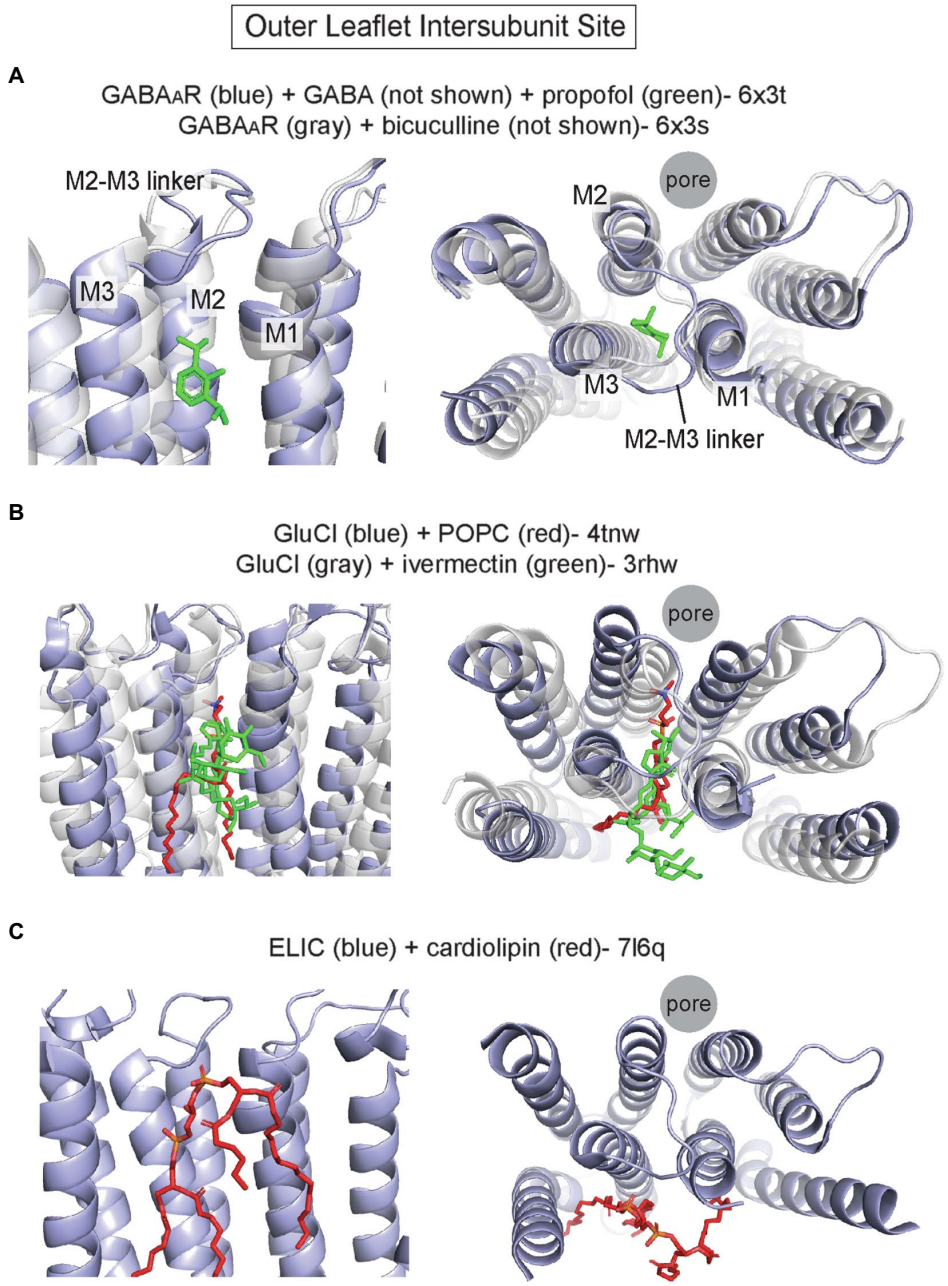
Outer leaflet intersubunit binding site in pLGICs. **(A)** α1/β2 interface of α1/β2/γ2 GABA_A_R with GABA and propofol (pdb 6x3t), and the α1/β2 interface of α1/β2/γ2 GABA_A_R with the competitive antagonist, bicuculline (pdb 6x3s). The activated conformation in the GABA + propofol structure is associated with an outward movement (away from the pore axis) of the top of M2, M3 and the M2-M3 linker. **(B)** GluCl with POPC (pdb 4tnw) and GluCl with ivermectin (pdb 3rhw). Binding of ivermectin to the intersubunit site opens the channel pore and is associated with similar conformational changes as noted above in the GABA_A_R. **(C)** Apo ELIC with cardiolipin (pdb 7l6q). The cardiolipin occupies a surface at the intersubunit site.

Perhaps the best characterized site for allosteric modulators in the TMD of pLGICs is an outer leaflet intersubunit site. Photolabeling studies and cryo-EM structures have definitively established this to be the binding site for the intravenous general anesthetics, etomidate, propofol, and pentobarbital, in heteromeric GABA_A_Rs (Li et al., 2006, 2010; Chiara et al., 2012, 2013; Jayakar et al., 2014; Kim et al., 2020) and nAchRs (Nirthanan et al., 2008; Hamouda et al., 2011; Jayakar et al., 2013; Figure 2A) with each drug showing specificity for certain interfaces. This site is also occupied by the positive allosteric modulator, ivermectin, in GluCl from *c. elegans* (Hibbs and Gouaux, 2011) and the α1 homopentameric GlyR (Kumar et al., 2020). The outer leaflet intersubunit site is located between M1 and M3 of neighboring subunits. The upper border of this site is formed by the M2-M3 loop, and more deeply bound ligands contact M2 (Figure 2A). In all pLGIC structures for which there are apo and agonist-bound states, this site undergoes major conformational changes with displacement and overall expansion of M1 and M3, and an associated outward shift of the M2-M3 linker that leads to opening of the pore-lining M2 (Basak et al., 2018a,b; Kumar et al., 2020; Noviello et al., 2021; Zhang et al., 2021b; Figure 2A). Thus, there is evidence to indicate that this outer leaflet intersubunit site is a conserved allosteric hotspot in pLGICs (Reynolds et al., 2011).

The first structure showing a phospholipid bound to this site is that of GluCl crystallized in the presence of POPC (palmitoyl-oleoyl phosphatidylcholine); a POPC density was identified in this site (i.e., the ivermectin binding site), which produced a significant shift of the TMD helices relative to a structure without POPC (Althoff et al., 2014). Compared to the ivermectin-bound structure of GluCl, which shows an open pore, the POPC-bound structure is non-conducting. Binding of the bulky ivermectin molecule to the intersubunit site leads to enlargement of this binding pocket, displacement of M3 and outward movement of the M2-M3 linker and M2: key conformational changes associated with opening of the channel pore (Figure 2B). Interestingly, POPS (palmitoyl-oleoyl phosphatidylserine) competes for ivermectin binding and potentiates agonist (glutamate) binding unlike POPC, suggesting that PS may positively modulate GluCl channel activity through this site. Unfortunately, structures of GluCl in the presence of POPS could not be obtained, and the modulatory effect of POPS on GluCl channel function is not known (Althoff et al., 2014). Nevertheless, the POPC-bound GluCl structure reveals how lipid binding to the outer leaflet intersubunit site may alter the conformation and activation state of a pLGIC.

Recent pLGIC structures determined by cryo-EM have also demonstrated lipid densities in this outer leaflet intersubunit site. Structures of the α1β2γ2 GABA_A_R in complex with the general anesthetics, propofol, and diazepam, show putative lipid densities in this site at each interface of this heteromeric channel (Kim et al., 2020). The structures were obtained from GABA_A_Rs in saposin-based nanodiscs with porcine brain polar lipids. The lipid densities vary in appearance at different subunit interfaces and are partially displaced or absent when the intersubunit sites are occupied by propofol (α1/β2) or diazepam (β2/α1 and γ2/β2). Interestingly, a phospholipid density is also observed at the α1/γ2 interface, which has been called an orphan site as no anesthetic is known to bind to this site (Nourmahnad et al., 2016). Structures of the homomeric 5-HT3aR in saposin-based nanodiscs with porcine brain lipids also show phospholipid-like densities in the outer leaflet intersubunit site (Zhang et al., 2021b). These densities are stronger in the agonist-bound structure compared to the apo structure and associated with significant expansion of the intersubunit pocket, suggestive that the phospholipid preferentially binds to the agonist-bound conformation of the channel. The bound phospholipid is buried between M1 and M3, making multiple hydrophobic interactions with these helices and the M2-M3 linker. Lastly, a recent apo structure of the prokaryotic pLGIC, ELIC, in SMA nanodiscs from *Escherichia coli* membranes shows cardiolipin bound to this site (Kumar et al., 2021; Figure 2C). The large cardiolipin molecule interacts with a region of the TMD that encompasses the outer leaflet intersubunit site, although the lipid is binding more to the surface of this site than in the aforementioned structures (Figure 2C). The observation of bound cardiolipin provides a glimpse into lipid interactions with ELIC in a membrane similar to its native lipid environment. However, only an apo structure was reported in this study limiting our understanding of lipid modulation, as cardiolipin increases the open probability of ELIC in the presence of agonist (Kumar et al., 2021). Whether cardiolipin or other phospholipids modulate ELIC channel activity through this site is not clear.

The outer leaflet intersubunit site has also been the subject of study using molecular dynamics (MD) simulations. Coarse-grained MD simulations of the GlyR in a neuronal-like membrane show increased binding of cholesterol at this site in the agonist-bound conformation compared to apo (Damgen and Biggin, 2021). The steroid intercalates between an enlarged space between M1 and M3 in the agonist-bound state, similar to results from MD simulations of the GABA_A_R that predict cholesterol binding in this site (Henin et al., 2014). The simulations also suggest that phospholipids interact differentially at this site; phospholipids form more stable interactions with pre-M1 in the agonist-bound state (Damgen and Biggin, 2021). Similarly, coarse-grained MD simulations of the muscle-type αβγδ nAchR in neuronal-like membranes show that cholesterol binds to the outer leaflet intersubunit site with the highest affinity, while n-3 polyunsaturated phospholipids also occupy this site with lower affinity (Sharp and Brannigan, 2021). Consistent with these computational studies, a crystal structure of a GLIC-α1GABA_A_R chimera shows densities of the cholesterol analogue, cholesteryl hemisuccinate, at approximately this site (Laverty et al., 2017).

In summary, the outer leaflet intersubunit site in pLGICs is a key binding site for allosteric modulators. Structural and computational studies indicate that phospholipids and steroids bind to this site, with certain lipids possibly favoring agonist-bound, activated conformations.

### Inner Leaflet Intersubunit Site

**Figure 3:**
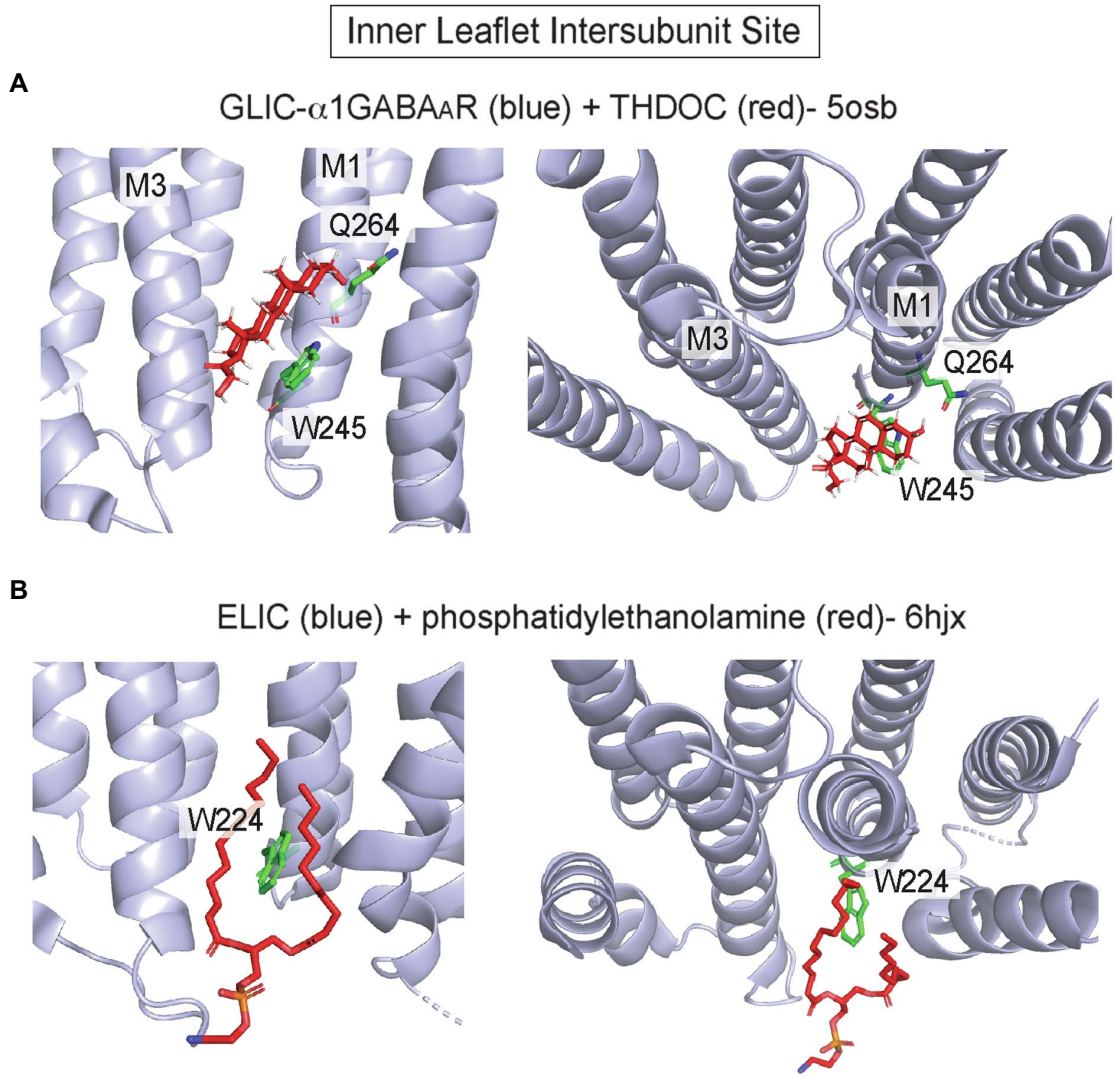
Inner leaflet intersubunit binding site in pLGICs. **(A)** GLIC-α1GABA_A_R chimera at pH 4.5 with THDOC (pdb 5osb). Q264 forms a hydrogen bond interaction with the 3-hydroxyl group of the neurosteroid, and W245 forms a hydrophobic ring stacking interaction with the steroid backbone. **(B)** Apo ELIC with phosphatidylethanolamine (pdb 6hjx). Shown is W224, which is a highly conserved tryptophan in pLGICs that interacts with neurosteroids **(A)** and forms this lipid binding site.

In pLGICs, the inner leaflet of the intersubunit groove also forms a hydrophobic pocket for allosteric modulators, especially neurosteroids. 3α-hydroxy-pregnane neurosteroids such as THDOC and the neurosteroid-analog anesthetic, alphaxalone, potentiate the GABA_A_R by binding to this site at the α1/β3 interface (Laverty et al., 2017; Miller et al., 2017; Chen et al., 2018, 2019; Sugasawa et al., 2019, 2020; Jayakar et al., 2020). The site is formed by residues that are generally conserved in pLGICs, including W245 and Q241 (this glutamine is conserved among α isoforms; Hosie et al., 2009) in M1 of α1, and L297, F301 and Y304 in M3 of β3. A hydrogen bond interaction between α1 Q241 and the neurosteroid 3-hydroxyl, and a ring stacking interaction between α1 W245 and the steroid backbone are critical for neurosteroid binding orientation and effect (Chen et al., 2019; Sugasawa et al., 2019; Figure 3A). Still, the exact mechanism by which neurosteroids modulate GABA_A_R gating is unclear as structures with and without bound neurosteroid show minimal changes in protein conformation, and in all structures the pore is in a putative desensitized conformation (Laverty et al., 2017; Miller et al., 2017; Chen et al., 2018). The inner leaflet intersubunit site is also thought to mediate volatile anesthetic modulation of pLGICs (Spurny et al., 2013).

In addition to being a critical site of action for anesthetics, such as alphaxalone, the inner leaflet intersubunit site is also a binding site for cholesterol and phospholipids. Photolabeling and docking studies show that cholesterol binds to this site in GLIC (Budelier et al., 2019) and the GABA_A_R (Lee, 2021), competing with neurosteroids. Moreover, cryo-EM structures of the GABA_A_R (Zhu et al., 2018) and nAchR (Walsh et al., 2018) show putative CHS densities in approximately the same site. In a crystal structure of ELIC in the apo/resting state, a phospholipid is also bound to this site (Henault et al., 2019; Figure 3B). The identity of this co-purified phospholipid is not established and may be PE (Henault et al., 2019) or PG (Tong et al., 2019; Sridhar et al., 2021). Interestingly, PG stabilizes the open state of ELIC relative to the desensitized state (Tong et al., 2019), and desensitization in ELIC is associated with a conformational change of M4 that may occlude this phospholipid binding site, providing a plausible mechanism for PG modulation of the open-desensitized equilibrium in ELIC (Henault et al., 2019). However, without an open structure of ELIC complexed to phospholipid, such a mechanism remains uncertain. Thus, the inner leaflet intersubunit site is an established binding site for allosteric modulators such as neurosteroids and may also be a site through which cholesterol or phospholipids modulate pLGIC gating.

### Intrasubunit Sites

**Figure 4:**
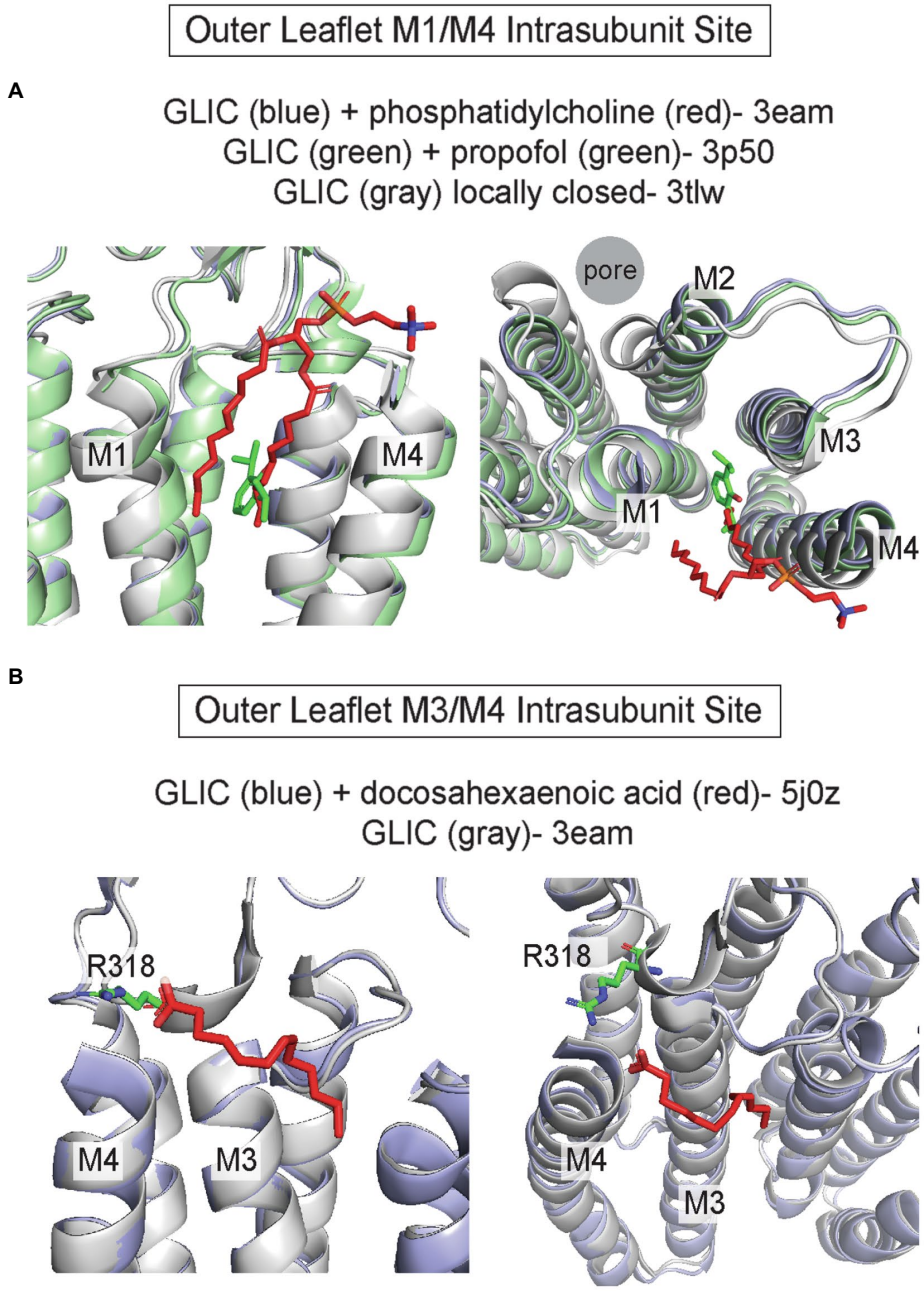
Outer leaflet intrasubunit binding sites in pLGICs. **(A)** GLIC with phosphatidylcholine (pdb 3eam), GLIC with propofol (pdb 3p50), and the GLIC loop 2–21′ oxidized mutant which is a locally-closed conformation (pdb 3tlw). The +phosphatidylcholine and + propofol structures are similar while the locally-closed structure shows a tilting of the top of M2, M1 and M4 that reduces the size of the intrasubunit space between the helical bundles. **(B)** GLIC with docosahexaenoic acid (DHA; pdb 5j0z), which is similar to the structure of GLIC without DHA (pdb 3eam). R118 in the β6-β7 loop likely forms an ionic interaction with the DHA carboxylate group.

Intrasubunit pockets between M1/M4 and M3/M4, particularly in the outer leaflet, are also binding sites for allosteric modulators and lipids. Propofol and the volatile anesthetic, desflurane, bind to an intrasubunit site in GLIC located within the four-transmembrane helix bundle; binding to this site is thought to mediate a potentiating effect (Nury et al., 2011; Arcario et al., 2017; Fourati et al., 2018; Figure 4A). MD simulations indicate that propofol and desflurane enter this site through a membrane-embedded pathway between M1 and M4 (Arcario et al., 2017). Propofol also binds to this intrasubunit pocket in ELIC (Kinde et al., 2016), the δ subunit of the *Torpedo* nAchR (Jayakar et al., 2013), and the β3 subunit of the GABA_A_R (Yip et al., 2013) based on photolabeling studies. The binding affinity of propofol is >20x higher for the agonist-bound/desensitized state compared to the apo state in the nAchR δ intrasubunit site, supporting the idea that propofol stabilizes the desensitized state of the nAchR through this site (Arevalo et al., 2005; Jayakar et al., 2013). 3α-pregnane neurosteroids such as allopregnanolone also bind to outer leaflet M3/M4 and M1/M4 intrasubunit sites in the GABA_A_R leading to distinct modulatory effects; allopregnanolone potentiates the GABA_A_R through an α1 M1/M4 intrasubunit site and inhibits through a β3 M3/M4 intrasubunit site (Sugasawa et al., 2020). Unlike propofol, these sites are located along the TMD-lipid surface within intrasubunit grooves. Similarly, the 5-HT3aR positive allosteric modulator, TMPPAA, may also bind to the M1/M4 intrasubunit groove (Polovinkin et al., 2018).

Structural studies have identified phospholipids, fatty acids, and cholesterol bound to these outer leaflet intrasubunit grooves in sites overlapping with that of allosteric modulators. One notable example is structures of GLIC where a phospholipid density occupies the outer leaflet M1/M3 intrasubunit site and is displaced when propofol is bound (Bocquet et al., 2009; Nury et al., 2011; Figure 4A). Interestingly, this lipid density is present in the GLIC open structure, but absent in locally-closed structures in which a shift in the top of M2, M3, and M4 reduces the volume of this intrasubunit site (Prevost et al., 2012; Figure 4A). This result suggests that phospholipid occupancy of this site stabilizes the open state of GLIC. On the other side of M4, the outer leaflet M3/M4 intrasubunit site is a binding site for the polyunsaturated fatty acid, docosahexaenoic acid (DHA), in GLIC (Basak et al., 2017; Dietzen et al., 2021; Figure 4B). The carboxylate group of DHA is adjacent to R318 forming an ionic interaction with this residue; however, it is less clear how the polyunsaturated tail interacts with the channel as this was not fully resolved in the structure (Figure 4B). The DHA binding site in GLIC is similar to the inhibitory neurosteroid binding site in the GABA_A_R (Sugasawa et al., 2020). Thus, the M3/M4 intrasubunit site may be a common binding site for DHA and neurosteroids that mediate inhibition of some pLGICs. Cholesterol binding to outer leaflet intrasubunit sites was first proposed based on cryo-EM structures of the *Torpedo* nAchR from native membranes in which large gaps between protein densities suggested the presence of lipids (Toyoshima and Unwin, 1988; Unwin, 1995, 2005; Miyazawa et al., 2003). Subsequent docking and MD simulations revealed that these sites are occupied by multiple cholesterol molecules, which are necessary to maintain the spaced-apart arrangement of transmembrane helices as well as contacts between ECD and TMD loops (Brannigan et al., 2008). Further refinements of the cryo-EM images of the nAchR in *Torpedo* membranes showed low-density patches corresponding to cholesterol at outer leaflet M1/M4 intrasubunit sites, particularly in the δ subunit (Unwin, 2017, 2020). Cholesterol occupancy of these sites is thought to underlie the requirement of this steroid for nAchR gating efficacy.

**Figure 5:**
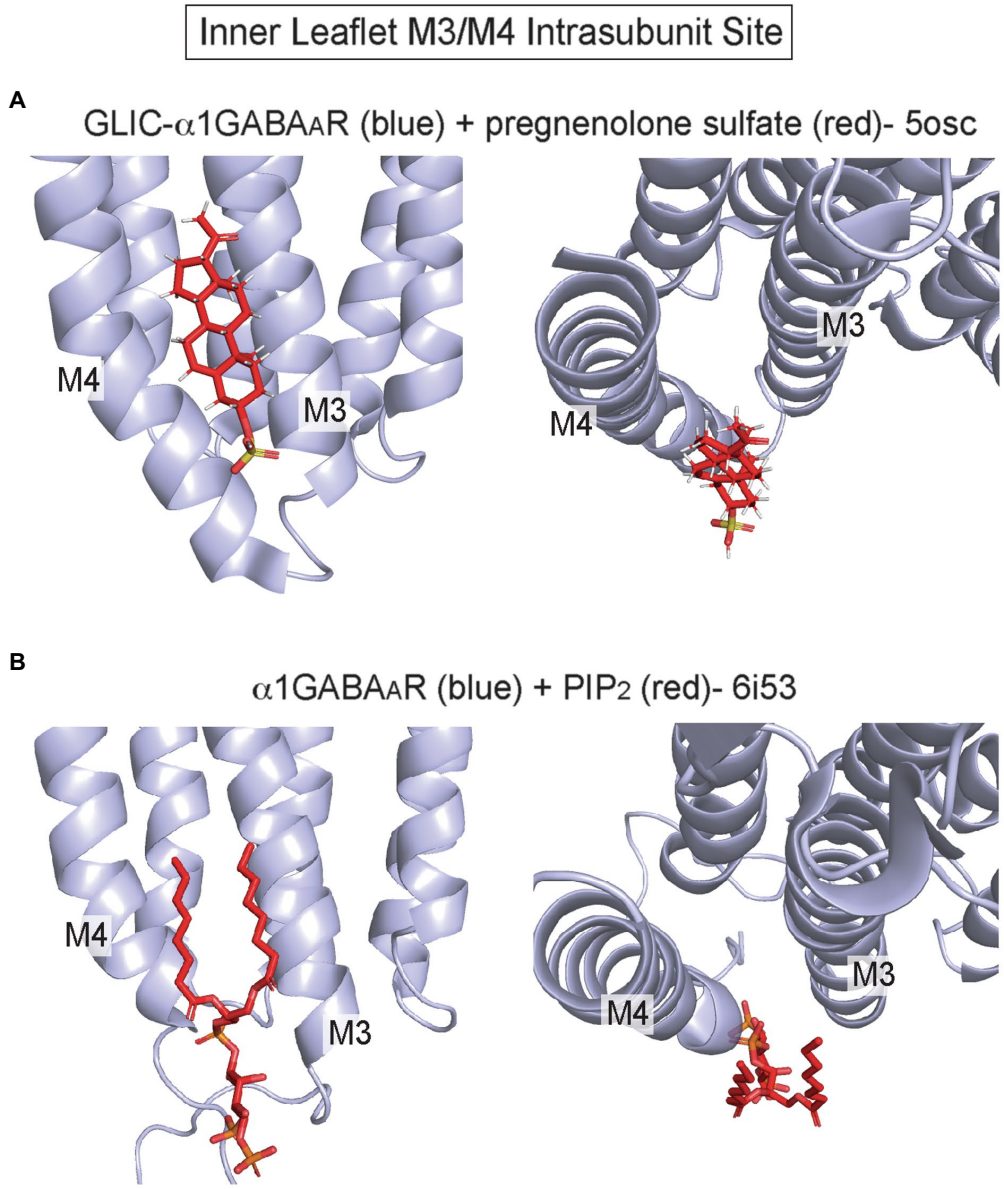
Inner leaflet M3/M4 intrasubunit binding site **(A)** GLIC-α1GABA_A_R with pregnenolone sulfate (pdb 5osc). **(B)** α1β3γ2 GABA_A_R with PIP_2_ bound to the α1 subunit (pdb 6i53).

Recent structures have also implicated an inner leaflet intrasubunit site between M3 and M4 as a lipid binding site, although there is little evidence to show that other allosteric modulators or drugs act at this site (Figure 1). The crystal structure of a GLIC-α1GABA_A_R chimera in complex with pregnenolone sulfate (PS) shows a density at this site consistent with PS (Laverty et al., 2017; Figure 5A). There is, however, some controversy whether PS inhibition of the GABA_A_R is mediated by this inner leaflet site, since this anionic neurosteroid is a rapid and effective inhibitor when applied extracellularly (Germann et al., 2019). This site may also be a cholesterol binding site based on a recent photolabeling study (Krishnan et al., 2021). Cryo-EM structures of the GABA_A_R and GlyR also show phospholipid densities in this site. In α1β3γ2 GABA_A_R structures from receptors in brain lipid nanodiscs, a density consistent with PIP_2_ is present solely in the α1 subunit at this intrasubunit site (Laverty et al., 2019; Figure 5B). The PIP_2_ density is present in the apo structure and structures complexed with biccuculine or GABA/alphaxalone, showing no obvious dependence on channel conformational. Moreover, GABA_A_R gating is not significantly impacted by PIP_2_; thus, the functional significance of this PIP_2_ binding site remains unknown. In GlyR structures from receptors in asolectin lipid nanodiscs, a phospholipid-like density is also apparent at this site in only the apo structure (Kumar et al., 2020).

In summary, outer leaflet intrasubunit sites in pLGICs are sites of action for anesthetics and other allosteric modulators, and phospholipids and cholesterol likely exert significant modulatory effects by binding to these sites. In contrast, the inner leaflet M3/M4 intrasubunit site is a recently established binding site for PIP_2_ and possibly other lipids; the functional significance of this site is unclear and may involve effects on ion channel trafficking instead of gating (Laverty et al., 2019).

## Lipid-Drug Binding Sites in Trp Channels

TRP channels are a superfamily of cation-selective channels that are implicated in many physiologic functions including temperature sensation and regulated by diverse stimuli including lipids (Clapham et al., 2001). Various TRP channels are regulated by PIP_2_ and other anionic phospholipids, cholesterol, neurosteroids, fatty acids, endocannabinoids, and many lipid metabolites produced with tissue inflammation (Wagner et al., 2008; Sisignano et al., 2014; Rohacs, 2015; Taberner et al., 2015; Morales-Lazaro and Rosenbaum, 2017). Most TRP channels are regulated by PIP_2_ or other phosphoinositides, and the effects of phosphoinositides on different TRP channels vary and are quite complex. For example, both activating and inhibiting effects have been described for PIP_2_ in TRPV1, TRPV4, and TRPC channels, while only activating effects have been reported in other TRPV channels (e.g., TRPV5) and all TRPM channels (Rohacs, 2015). The recent explosion of TRP channel structures have revealed many lipid binding sites, some of which are also sites for exogenous drugs/modulators or phosphoinositides. We will review these structures with a focus on lipid-drug sites and PIP_2_ binding sites. For a more comprehensive treatment of ligand binding sites in TRP channels, the reader may refer to a recent review on this topic (Zhao et al., 2021).

**Figure 6:**
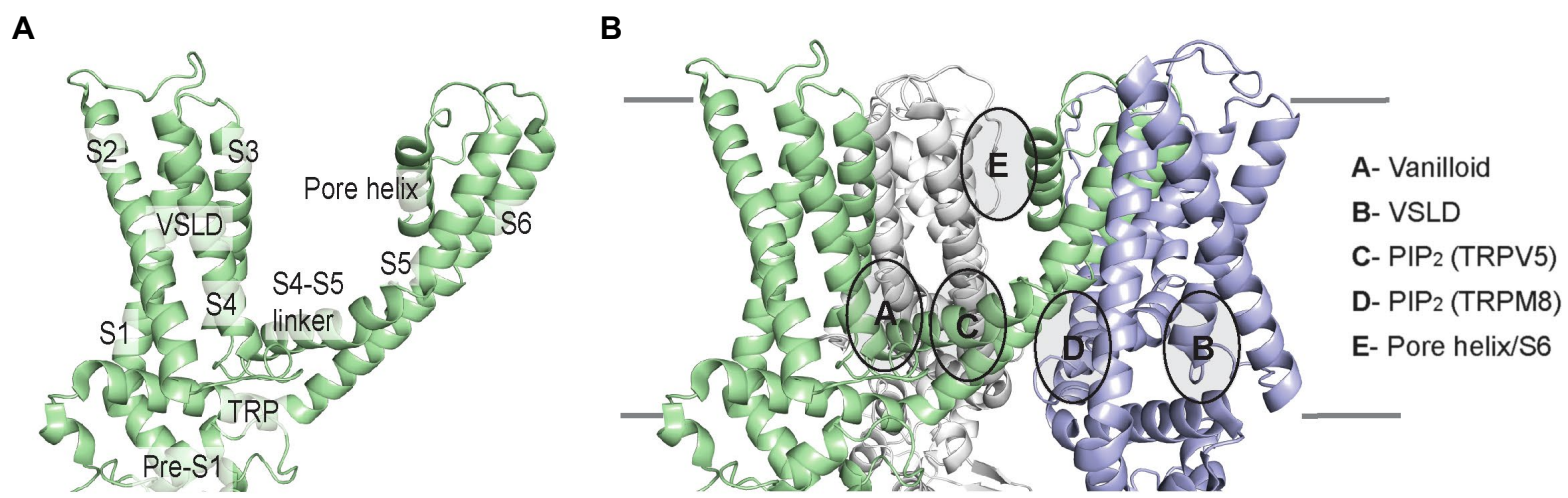
TRP channel TMD architecture, and schematic of lipid-drug or lipid binding sites in TRP channels illustrated using the TRPV1 lipid nanodisc structure (pdb 5irz). **(A)** Single subunit of TRPV1 labeling the helices and domains. The VSLD consists of S1-S4 helices. **(B)** Three subunits of the TRPV1 tetramer are shown indicating the location of lipid-drug or lipid binding sites as described.

The architecture of TRP channels consists of a tetramer with six transmembrane helices (S1–S6). These helices comprise the voltage-sensing like domain (VSLD, S1–S4) and pore domain (S5–S6), connected by the critical S4–S5 linker. Also, lining the inner membrane interface are the N-terminal pre-S1 domain and the C-terminal TRP domain (Figure 6A). Amidst these TMD elements are semi-conserved inner and outer leaflet ligand-binding pockets that in many TRP channels are also lipid binding sites. We will examine these lipid-drug binding sites, which include the vanilloid-binding site (i.e., the binding site for capsaicin in TRPV1) and the VSLD binding site, as well as binding sites for PIP_2_ and other lipids (Figure 6B).

### Vanilloid Binding Site

**Figure 7:**
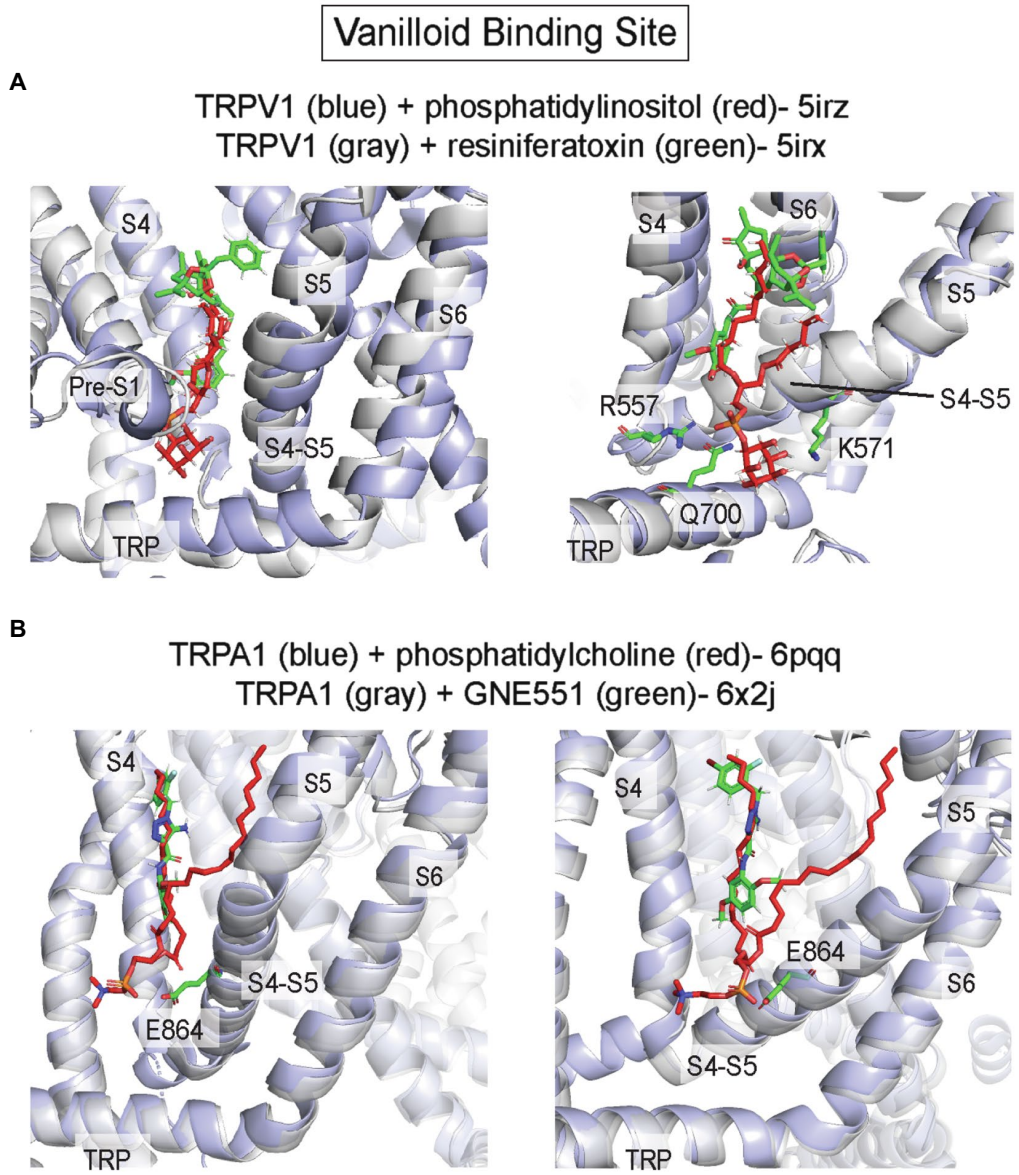
Vanilloid binding site in TRP channels. **(A)** TRPV1 with phosphatidylinositol (PI; pdb 5irz) and TRPV1 with resiniferatoxin (RTX; pdb 5irx). The headgroup of PI is predicted to interact with R557, K571 and Q700. Replacement of PI in this pocket with RTX leads to outward movement of the S4-S5 linker, TRP domain and S6 producing opening of the channel pore. **(B)** TRPA1 with phosphatidylcholine (pdb 6pqq) and TRPA1 with GNE551 (pdb 6x2j). E864 in the S4-S5 linker is predicted to interact with the phospholipid headgroup. There is no difference in protein conformation between lipid-bound and agonist-bound structures.

The vanilloid binding site, which binds capsaicin (the active component of chili peppers) in TRPV1, is an inner leaflet pocket bound by S3/S4 of the VSLD, the S4-S5 linker, and the pore-forming S6 of the adjacent subunit (in domain-swapped TRP channels such as TRPV1; Cao et al., 2013; Gao et al., 2016; Figures 6, 7). A structure of TRPV1 complexed with the vanilloid agonist, resiniferatoxin (RTX), shows specific interactions of RTX in this hydrophobic pocket that are associated with outward displacement of the S4–S5 linker and S6, and opening of a lower pore gate at S6 (Gao et al., 2016; Figure 7A). The vanilloid binding site is also the site of action of the TRPV5 antagonist, econazole (Hughes et al., 2018a), and the TRPA1 biased agonist, GNE551 (Liu et al., 2021; Figure 7B). Moreover, mutations within this site in TRPV2 and TRPV3 render these channels sensitive to vanilloid agonists (Yang et al., 2016; Zhang et al., 2019). Thus, this site is relatively conserved among all TRP channels and is an allosteric hotspot, where conformational changes of the S4-S5 linker and TRP domain are linked to the pore-lining S6 (Zhao et al., 2021).

It is no surprise, then, that the vanilloid binding site is often occupied by a lipid, especially in cryo-EM structures of TRP channels in lipid nanodiscs. The most notable example is a PI (phosphatidylinositol) phospholipid that was resolved in the apo, closed structure of TRPV1 in asolectin nanodiscs (Gao et al., 2016). Unlike the vanilloid agonist, RTX, the anionic PI headgroup interacts with a slightly expanded binding pocket that includes polar/charged interactions with residues at the pre-S1 region and TRP domain, in addition to the bottom of S4 and the S4-S5 linker (Figure 7A). Specifically, the PI headgroup interacts with R557 and K571 in the S4-S5 linker (Figure 7A). When a vanilloid binds, this pocket re-arranges such that R557 interacts with E570 within the S4-S5 linker pulling the S4-S5 linker, S5 and S6 away from the central axis and opening the channel pore (Figure 7A). A recent cryo-EM study showing multiple snapshots of TRPV1 demonstrates stepwise displacement of the PI acyl chains by RTX followed by the PI headgroup (Zhang et al., 2021a). Visualization of these intermediates shows that PI lipids from all four subunits must be displaced to produce conformational changes in S5 and S6 associated with channel opening. The striking overlap between PI and RTX in this site suggests the possibility that PI is an antagonist of vanilloids in TRPV1. Indeed, a recent study showed that PI inhibits TRPV1 in excised inside-out patches, and that inhibition is the strongest at low capsaicin concentrations and lost at high capsaicin concentration, suggesting that PI competitively inhibits capsaicin activation of TRPV1 (Yazici et al., 2021). A similar example of overlapping lipid-drug binding modes was also observed in TRPA1 structures in complex with the agonist, GNE551 (Suo et al., 2020; Liu et al., 2021; Figure 7B). A phospholipid occupies this site (equivalent to the vanilloid binding site in TRPV1) in the TRPA1 apo structure and is displaced when GNE551 binds. The phospholipid headgroup is adjacent to E864 in the S4-S5 linker (Figure 7B) and mutation of this residue to a bulky tryptophan, which may occlude lipid binding to this site, increases basal channel activity (Liu et al., 2021). This result is consistent with lipid binding to this site antagonizing channel activation. However, the apo and GNE551-bound structures show a similar non-conducting conformation, and so the mechanism of GNE551-mediated activation of TRPA1 is not entirely clear (Figure 7B). Also, it is not known whether specific phospholipids bind to this site in TRPA1 or if lipids antagonize GNE551-mediated TRPA1 activation.

A lipid occupies the equivalent vanilloid binding site in many other TRP channel structures, some of which show evidence of state-dependent lipid binding. Recent structures of TRPV3 in NW11 asolectin nanodiscs at different temperatures illuminate the mechanism of heat activation of TRPV3. The closed structure shows a phospholipid in this site that is excluded from this pocket by a conformational change in the heat-activated open structure (Nadezhdin et al., 2021), similar to the state-dependence of PI binding in TRPV1. MD simulations suggest that this pocket is relatively non-selective for different glycerophospholipids (PI, PC, PS, and PE), and is less favorable for cholesterol (Nadezhdin et al., 2021). In contrast, structures of TRPV6 and TRPV3 in MSP2N2 asolectin nanodiscs show a phospholipid density in this site in activated conformations with loss of the lipid in the closed conformations, suggesting a mechanism whereby lipids may be agonists or positive allosteric modulators of these channels (McGoldrick et al., 2018; Deng et al., 2020). Structures of other TRP channels including TRPV2 (Zubcevic et al., 2016), TRPM2 (Zhang et al., 2018), TRPM4 (Autzen et al., 2018; Duan et al., 2018b), TRPC4 (Duan et al., 2018a), TRPC5 (Duan et al., 2019), TRPC6 (Bai et al., 2020), and the yeast TRPY1 (Ahmed et al., 2021) also show phospholipid or CHS-like densities in this site. Endocannabinoids, such as anandamide and N-arachidonoyl dopamine, are also thought to activate TRPV1 by binding to this site (Li et al., 2021). Thus, the vanilloid binding site is likely an important lipid binding site in most TRP channels; differences in state-dependent lipid binding as revealed from cryo-EM structures suggest distinct effects of lipids in regulating TRP channel structure and function.

### VSLD Binding Site

In many TRP channels, the VSLD, which comprised the S1–S4 helix bundle, forms an inner leaflet ligand binding pocket (Figure 6). Ligands that act through this site include the TRPM8 agonists, menthol and icilin (Yin et al., 2018, 2019), the TRPC6 inhibitor, AM-1473 (Bai et al., 2020), the TRPV3 inhibitor, osthole (Neuberger et al., 2021), and the TRPV6 inhibitor, 2-Aminoethoxydiphenyl borate (2-APB; Singh et al., 2018). When 2-APB binds to this site in TRPV6, the associated conformational change at the bottom of S3 and the S4–S5 linker upwardly displaces a nearby lipid in the VSLD binding site and is associated with closure of the lower gate in the pore (Singh et al., 2018). A putative activating lipid in the vanilloid binding site is also displaced upward with 2-APB binding (Singh et al., 2018). In addition to TRPV6, other structures that also demonstrate lipid density in the VSLD binding site are TRPV1 (Gao et al., 2016), TRPV3 (Deng et al., 2020), and TRPV5 (Hughes et al., 2018a).

### PIP_2_ and Other Lipid Binding Sites

**Figure 8:**
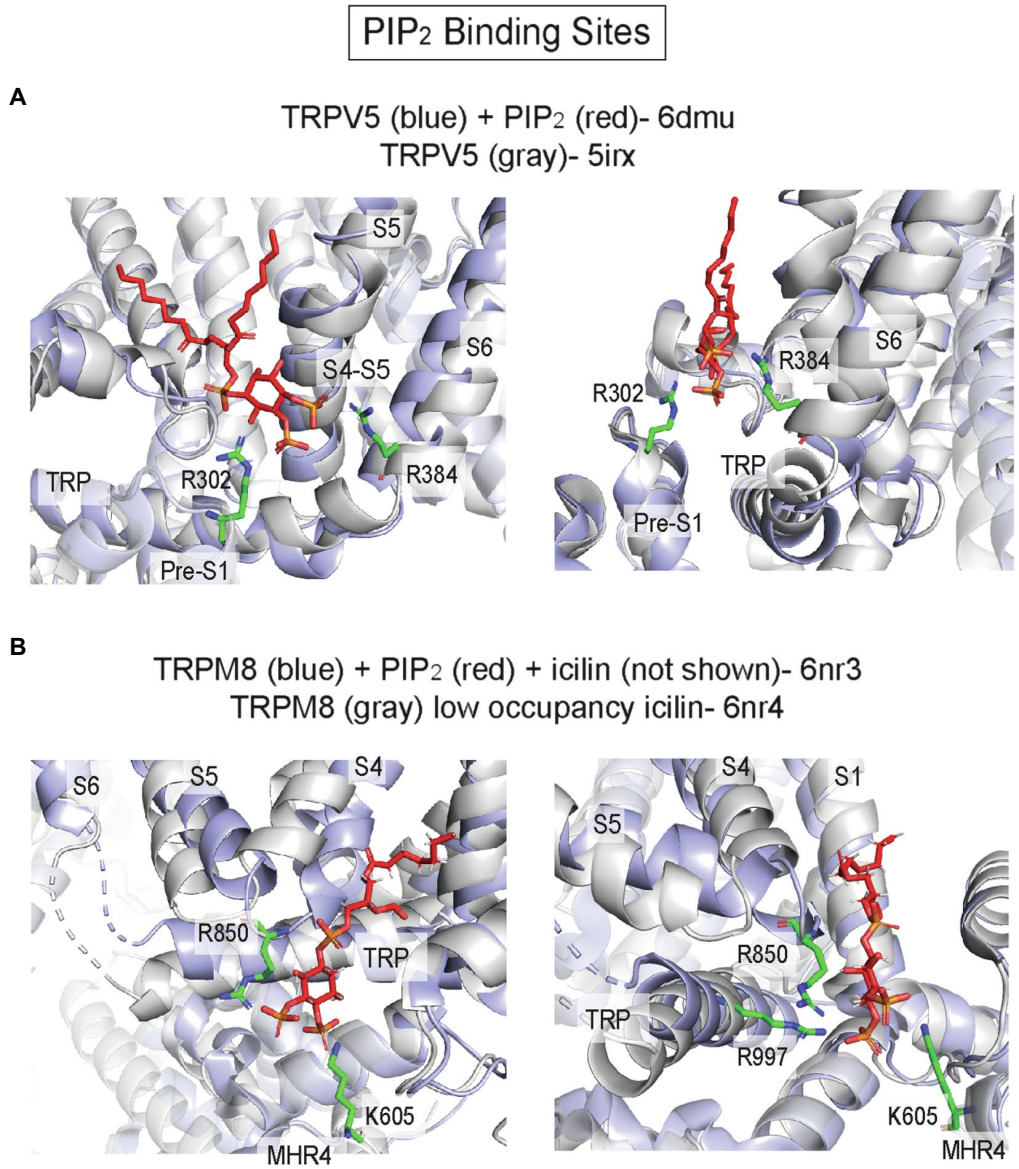
PIP_2_ binding sites in TRP channels. **(A)** TRPV5 with PIP_2_ (pdb 6dmu) and TRPV5 without PIP_2_ (pdb 5irx). R302 and R384 are predicted to interact with the PIP_2_ headgroup. Binding of PIP_2_ is associated with outward movement of the S4-S5 linker and S6 away from the pore axis. **(B)** TRPM8 with PIP_2_ and icilin (pdb 6nr3) and TRPM8 with low occupancy icilin (pdb 6nr4). K605, R850, and R997 are predicted to interact with the PIP_2_ headgroup. Binding of the agonist icilin with high occupancy (not shown in this image) is associated with tilting of the TRP domain and movement of S4 (S4b) and S5, which brings R850 and R997 near the PIP_2_ headgroup.

Most TRP channels are regulated by PIP_2_, a major phosphoinositide in the plasma membrane inner leaflet. Both positive and negative regulatory effects of PIP_2_ have been reported in TRPV1 and other TRP channels, and there is evidence to suggest that multiple distinct binding sites account for these varied effects (Yazici et al., 2021). While phosphoinositides, such as PI antagonize TRPV1 activation by competing for vanilloid agonist binding, another site(s) likely mediates PIP_2_ activation of TRPV1. Molecular docking and MD simulations identified a putative PIP_2_ binding site in TRPV1, adjacent to the vanilloid binding site, where the headgroup is interacting with basic residues in the S4-S5 linker and TRP domain (Poblete et al., 2015; Yazici et al., 2021). Mutations of these residues significantly right-shift the PIP_2_ dose–response curve (Poblete et al., 2015). Strikingly, a cryo-EM structure of TRPV5 in MSP2N2 asolectin nanodiscs with diC8-PIP_2_ shows a PIP_2_ headgroup bound to approximately the same inner leaflet site as was predicted for TRPV1, interacting with basic residues (R302 and R384) in the S4–S5 linker and the N-linker before the pre-S1 helix (Figures 6, 8A; Hughes et al., 2018b). The structure demonstrates how PIP_2_ binding produces an outward shift of the S4-S5 linker, S6 and the TRP domain (Figure 8A), which leads to widening of the lower gate and the selectivity filter. MD simulations also show that PIP_2_ stably binds to this site in TRPV6 (Hughes et al., 2018b). Thus, this site may mediate PIP_2_ activation of multiple TRP channels in the vanilloid subfamily including TRPV1, TRPV5, and TRPV6.

Cryo-EM structures of TRPM8 in complex with PIP_2_ reveal a distinct inner leaflet PIP_2_ binding site, where the headgroup interacts with the pre-S1 domain, TRP domain, S4-S5 linker and MHR4 (melastatin homology region; Figures 6, 8B; Yin et al., 2019). Compared to the TRPV5 PIP_2_ binding site, this site is located on the opposite side of the VSLD and the TRP domain (Figure 6). The structure of TRPM8 complexed with PIP_2_ and the agonist, icilin, demonstrates allosteric coupling between these ligands: high occupancy of icilin in the VSLD inner leaflet site is associated with an upward tilt of the TRP domain and movement of S4 and S5 that enhances interactions between PIP2 and key residues such as R850 in S5 and R997 in the TRP domain (Yin et al., 2019; Figure 8B). It is not known if this novel site is a PIP_2_-specific binding site in other TRP channels; nevertheless, multiple TRP channel structures show phospholipid or CHS-like densities at this site including TRPM4 (Autzen et al., 2018; Duan et al., 2018b), TRPC3 (Fan et al., 2018), TRPC4 (Duan et al., 2018a), TRPC5 (Duan et al., 2019), and TRPA1 (Suo et al., 2020).

In addition to inner leaflet lipid binding sites, multiple lipid densities are often present at outer leaflet sites in TRP channel structures (Gao et al., 2016; Autzen et al., 2018; Deng et al., 2020). The most commonly observed site is located between the pore helix and outer portion of S6 of the adjacent subunit (Figure 6), and structures of some TRP channels suggest that lipid binding at this site is state-dependent. For example, the structures of TRPV3 in MSP2N2 asolectin nanodiscs show a phospholipid density at this site that shifts its binding mode with channel activation as the pore helix moves downward opening the selectivity filter (Deng et al., 2020). Structures of TRPM8 in amphipols show a CHS-like density at this site in the desensitized conformation that is absent in the closed conformation, due to a large conformational change in the outer pore loop and pore helix that occludes this site (Diver et al., 2019). Structures of TRPM4 (Autzen et al., 2018; Duan et al., 2018b), TRPC3 (Fan et al., 2018), TRPC4 (Duan et al., 2018a), TRPC5 (Duan et al., 2019), and TRPC6 (Bai et al., 2020) also show a phospholipid or CHS-like density at this site. Multiple TRP channels are positively or negatively regulated by steroids (Morales-Lazaro and Rosenbaum, 2017), including pregnenolone sulfate (PS) activation of TRPM3 (Wagner et al., 2008; Uchida et al., 2016) and testosterone activation of TPRM8 (Asuthkar et al., 2015a,b). Interestingly, TRPM3 activation by pregnenolone sulfate is only observed with extracellular application of this anionic neurosteroid, meaning that it is likely acting through an outer leaflet binding site. The outer leaflet pore helix/S6 lipid binding site could be the site of action for PS in TRPM3, although this remains to be tested and there is no available structure for this channel.

## Discussion

Two interesting observations arise from examining bound lipids in high-resolution structures of pLGICs and TRP channels. First, many of these lipid binding sites are known sites for exogenous ligands/drugs. Notable examples are the binding site for anesthetics in the outer leaflet intersubunit pocket in the GABA_A_R or the vanilloid binding site in TRPV1. Second, the sites of lipid binding, even those which are not shared by other ligands, are often conserved among different pLGICs or TRP channels. While it remains unclear in most cases whether lipid occupancy of these sites is functionally important, the above observations suggest that these sites are likely allosteric hotspots in the transmembrane domain of these proteins (Reynolds et al., 2011), and promising targets for structure-based drug design. Indeed, structure-based virtual screening of the econazole binding pocket in TRPV5, which is equivalent to the vanilloid binding site in TRPV1, yielded several high affinity and specific inhibitors of TRPV5 (Hughes et al., 2019). The binding pocket of a lipid may be overlapping but non-identical to that of a drug at the same site such that unique lipid-protein interactions may contribute to its binding and effect. For example, PI interacts with residues in the vanilloid binding pocket that are not shared with RTX or capsaicin (Gao et al., 2016), and mutation of these residues alter PI inhibition and vanilloid agonist sensitivity in TRPV1 (Yazici et al., 2021). As drug design targeting the lipid-protein interface gains interest (Payandeh and Volgraf, 2021), understanding the determinants of lipid binding and regulation of ion channels may facilitate this effort.

The finding that many hydrophobic drugs/ligands share binding sites with lipids also raises the hypothesis that the cellular lipid environment influences the potency of these drugs through competitive interactions with lipids. Such a hypothesis is difficult to test and has seldom been demonstrated. However, there are a few examples among TRP channels and pLGICs that suggest interactions between lipids and ligands. These include PI inhibition of capsaicin activation of TRPV1 (Yazici et al., 2021), PS inhibition of ivermectin binding in GluCl (Hibbs and Gouaux, 2011), or cholesterol inhibition of neurosteroid potentiation of the GABA_A_R (Sooksawate and Simmonds, 2001b). In the latter case, epi-cholesterol (the 3-hydroxy diastereomer of cholesterol) does not have the same effect as cholesterol, arguing for a direct/specific interaction. Although it is still difficult to rule out an allosteric mechanism of cholesterol inhibition of neurosteroid effect, the fact that cholesterol and neurosteroids compete for common sites in pLGICs (Budelier et al., 2019; Lee, 2021) supports a competitive mechanism. There are also differences in general anesthetic potency that may be a consequence of changes in lipid environment. For example, diet-induced depletion of rat brain ω-3 and ω-6 fatty acid content significantly increases sensitivity to volatile anesthetics (Evers et al., 1986). Moreover, volatile anesthetics are less potent in infants, and more potent in neonates, the elderly and pregnant women. While there could be a multitude of factors that contribute to these differences (Moody et al., 2021), it is plausible that changes in neuronal lipid membrane content with age or different physiologic states contributes to anesthetic sensitivity by modulating GABA_A_R function. Lipids may alter anesthetic sensitivity through competitive or non-competitive mechanisms. As our molecular and cellular understanding of lipid regulation of ion channels advance, it may be feasible to test these ideas directly.

## Knowledge Gaps and Future Developments

The availability of cryo-EM structures of ion channels with bound lipids is a critical starting point to understanding structural mechanisms of lipid regulation. For most of these structures, it is unknown whether these sites are selective for certain lipids and whether lipids regulate channel function by binding to these sites. With structure determination accelerating, two major knowledge gaps need to be addressed concurrently: (1) understanding the exact effects of lipids on ion channel function and (2) determining lipid binding affinities at specific sites in different conformational states. Such understanding taken together will be necessary to answer the essential question of whether certain lipids regulate ion channel function through a specific site(s) or through indirect effects on lipid bilayer properties. We highlight several approaches and suggest new developments to these techniques to address the above knowledge gaps.

### Functional Measurements of Lipid Regulation

Lipid regulation of ion channel function is best determined in model membranes where lipid composition can be precisely controlled. The necessity of reconstituting biochemical quantities of pure, functional protein has made such experiments challenging and mostly limited to prokaryotic ion channels. However, advances in large-scale expression of mammalian membrane proteins (Goehring et al., 2014) and solubilization/reconstitution in superior detergents or nanodisc scaffolds (Chae et al., 2012; Dorr et al., 2014; Winterstein et al., 2018; Yu et al., 2021) should enable more functional studies of mammalian ion channels in model membranes. Ion channel function can be assessed in planar lipid bilayers, giant liposomes by excised patch-clamp, or small unilamellar liposomes with tracer flux measurements. While the former two permit measurements of single channel currents, the latter provides the most versatile system for reconstitution in a wide range of lipid environments including asymmetric bilayers (Doktorova et al., 2018). Recently, measurements of transporter function in stably asymmetric liposomes were reported (Markones et al., 2020). It is conceivable that established methods to manipulate phospholipid content in the outer leaflet of liposomes (Markones et al., 2018, 2020) could be interfaced with ion channel flux assays in proteoliposomes. Activating ligand-gated ion channels from the extra-liposomal space would guarantee that only the activity of channels of one orientation would be measured, ensuring the relevance of the asymmetric bilayer. Such an approach could be used to mimic native-like asymmetric lipid environments. More interestingly, it could also be used to definitively determine if a specific lipid regulates ion channel function from the inner or outer leaflet, clarifying whether an inner or outer leaflet site mediates the effect. Asymmetric planar lipid bilayers or the recently developed contact bubble bilayer were used to demonstrate that anionic phospholipids activate the prokaryotic potassium channel, KcsA, and viral potassium channels from the inner leaflet (Iwamoto and Oiki, 2013, 2015; Winterstein et al., 2021). However, unlike liposomes in which methods such as zeta potential measurements are available to verify generation and stability of an asymmetric bilayer (Markones et al., 2018), no such verification procedure is routinely applied for asymmetric planar bilayers or contact bubble bilayers such that one is less certain if inter-leaflet mixing or protein-induced scrambling of lipids takes place in these systems (Montal and Mueller, 1972).

Functional studies of ligand-gated ion channels also require the capability to examine gating kinetics, including processes such as rapid activation in response to agonist and slow desensitization in the continued presence of agonist. This can be achieved with a stopped-flow fluorometric ion flux assay (Posson et al., 2018), which measures the rate of fluorescence quenching of a fluorophore as a quenching ion (usually thallium) enters the liposomes through ion channels. This technique has been successfully applied to proton-gated (Rusinova et al., 2014), calcium-gated (Posson et al., 2015), cyclic nucleotide-gated (Schmidpeter et al., 2020), and pentameric ligand-gated ion channels (Menny et al., 2017; Tong et al., 2019). We anticipate that the stopped-flow ion flux assay, together with innovative methods to make asymmetric proteoliposomes, will provide novel insights into the mechanism of lipid regulation of ion channels.

### Measurements of Lipid Binding

Assessment of lipid binding sites and affinities, especially state-dependent binding, is a challenging but critical step to understand lipid regulation of ion channels through a direct binding mechanism. Crystallography and cryo-EM undoubtedly remain powerful approaches to identify lipid binding sites and protein conformational changes associated with bound lipids. Future studies will likely seek to obtain structures of a single ion channel reconstituted with different lipids in the same nanodisc scaffold; this may be a useful approach to systematically examine the specificity and structural effects of lipid binding. The presence and absence of lipids in different conformational states from such structures suggests state-dependent lipid binding. However, the absence of a resolved lipid density does not rule out occupancy of a specific lipid at a given site. An example of this is the case of neurosteroid binding to the GABA_A_R. While crystal structures only show neurosteroid binding to an inner leaflet intersubunit site (Laverty et al., 2017; Miller et al., 2017; Chen et al., 2018), photolabeling studies also indicate neurosteroid binding to outer leaflet M1/M4 and M3/M4 intrasubunit sites (Chen et al., 2019; Sugasawa et al., 2019, 2020). The absence of neurosteroids at these sites in the structures could be because the neurosteroid is highly flexible in these sites (multiple binding modes), or that binding favors a conformation not captured in the structures. It is also possible that the process of membrane protein crystallization or cryogenic freezing, or the use of detergents and nanodisc scaffolds alters the interaction of lipids with membrane proteins. Moreover, lipid densities from cryo-EM and crystallography studies may not provide the resolution to definitively distinguish one lipid species from another. For these reasons, photo-affinity labeling, combined with residue-level identification of labeled sites by mass spectrometry, offers a complementary approach for identifying lipid binding sites, and has been utilized to identify cholesterol and neurosteroid binding sites in membrane proteins (Budelier et al., 2017a,b; Cheng et al., 2018; Krishnan et al., 2021). A recent study also showed that photo-affinity labeling can be used to identify binding sites for the polyunsaturated fatty acid, DHA, in a pLGIC, and to assess state-dependent binding of DHA at these sites (Dietzen et al., 2021). The same strategy should be feasible for other lipids, such as sphingolipids (Dadsena et al., 2019), phospholipids and lysophospholipids (Xia and Peng, 2013; Peng et al., 2014), endocannabinoids (Dixon et al., 2012), or fatty acid metabolites for residue-level identification of binding sites. Developing new reagents with optimal photochemistry, especially the use of trifluoromethylphenyl diazirine (TPD) photoreactive groups, may be essential for this effort (Cheng et al., 2019; Dietzen et al., 2021; Krishnan et al., 2021). Once a site(s) is identified, competition photolabeling experiments with any lipid or ligand can be used to determine relative binding affinities at individual sites as well as state-dependent changes in binding affinity, as has been extensively applied for anesthetic binding sites in the GABA_A_R (Li et al., 2010; Chiara et al., 2013; Jayakar et al., 2014, 2019).

Other experimental methods to examine lipid binding affinities in ion channels include tryptophan fluorescence quenching by brominated lipids (Powl et al., 2003; Carney et al., 2007; Marius et al., 2008), a soluble lipid-binding assay using a fluorescently tagged channel and lipid with fluorescence or bioluminescence resonance energy transfer (FRET/BRET; Cabanos et al., 2017; Robinson et al., 2019), and native mass spectrometry (Laganowsky et al., 2013, 2014). These are useful methods to measure direct lipid binding and relative affinities and should also permit assessment of state-dependent lipid binding (Powl et al., 2005; Tong et al., 2019). However, they suffer from relatively low structural resolution, reducing the certainty that lipid binding is measured from a single site. While BRET or FRET R_o_ distances (distance of 50% energy transfer) range from 2 to 10 nm (Clegg, 1995; Weihs et al., 2020), the R_o_ for quenching of brominated lipids is ~8 Å providing the best resolution. Unfortunately, most ion channels have multiple tryptophans in the TMD, such that binding to a single site cannot be interrogated using the native channel. It may be possible to examine binding to single sites with brominated lipids by removing native tryptophans (Chen et al., 2010) or site-specific introduction of a fluorescent, unnatural amino acid such as ANAP (Puljung, 2021).

Molecular dynamics (MD) simulations, with unparalleled spatiotemporal resolution, have also been leveraged to characterize binding of lipids to a wide range of membrane proteins including ion channels (Kasimova et al., 2014; Tian et al., 2019; Buyan et al., 2020; Dietzen et al., 2021; Kapoor et al., 2021). A major hurdle is the mismatch between the timescales accessible to MD simulations and the rates of native lipid diffusion, limiting sampling of binding sites by lipid species. Efforts to reduce this mismatch are ongoing including development of hyper-specialized computational hardware and exploitation of graphics processing units (Salomon-Ferrer et al., 2013; Eastman et al., 2017; Kutzner et al., 2019; Phillips et al., 2020). Despite these advances, milliseconds is still the longest timescale available to all-atom MD simulations even with hyper-specialized hardware (Carnevale et al., 2021), which may not be sufficient to properly sample all protein-lipid interactions. Therefore, the use of dimension-reduced systems has become popular to study lipid binding (Ohkubo et al., 2012; Panahi and Feig, 2013; Souza et al., 2021). Dimension-reduced systems (i.e., coarse-grained, implicit solvent/membrane, united atom approach) improve protein-lipid sampling by reducing the number of calculations needed per time, allowing the simulation to reach longer timescales. The trade-off is that significant atomic-level detail is lost. In some cases (i.e., coarse-grained systems), this can be rectified by backmapping, or reverse coarse-graining, to obtain all-atom systems (Wassenaar et al., 2014). Such a strategy has been applied to improve protein-lipid sampling and achieve atomistic detail in studying lipid interactions with a pLGIC (Sridhar et al., 2021).

Enhanced sampling techniques may also be useful to improve modeling of the equilibrium ensemble of membrane protein systems (Bernardi et al., 2015; Yang et al., 2019). These techniques exploit the unique ability of simulation to selectively modify the energetic landscape of the system (Mori et al., 2013; Miao et al., 2020), and preferentially sample the microstates of the statistical ensemble that are desired (Zimmerman and Bowman, 2015; Zuckerman and Chong, 2017). These novel techniques have yet to be extensively applied to lipid binding in membrane proteins. However, a recent study showed that information obtained from enhanced sampling can be reweighted to determine the energetics and kinetics of protein-lipid interactions (Bhattarai et al., 2020). Lastly, classic free energy calculations are being adapted to study lipid binding to membrane proteins. Lipid binding sites present a unique challenge in free energy calculations in that they are generally superficial, and it is difficult to distinguish bound lipid from unbound lipid as it is both ligand and solvent. Changing the definition of a bound ligand/lipid (streamlined alchemical free energy perturbation, SAFEP) has allowed more accurate quantification of binding free energies and description of the non-ideality of cholesterol-GPCR interactions (Salari et al., 2018). This new approach promises to shed light on many protein-lipid interactions by calculating free energies of lipid binding to specific sites. Ideally, comparison of such free energy calculations with experimentally determined binding affinities would provide the most convincing application of these methods.

## Conclusion

High-resolution structures of ion channels, especially those embedded in lipid nanodiscs, show multiple lipid-drug binding sites in pLGIC and TRP channel families. While these sites are generally conserved among multiple members of each family, they are also the sites of action of some highly specific and potent ligands such as ivermectin in GluCl, capsaicin in TRPV1, and GNE551 in TRPA1. Thus, drug discovery efforts targeting these lipid binding sites in different channel subtypes may yield specific new modulators/inhibitors as was the case for TRPV5 (Hughes et al., 2019). Current understanding of how lipids regulate ion channel structure and function through these sites is far from complete and does not enable even qualitative predictions of lipid interactions with these proteins. Future research should focus on understanding the specificity and determinants of lipid binding to these sites in different functional states.

## Author Contributions

WWLC drafted the manuscript. MJA and JTP wrote and edited the manuscript. All authors contributed to the article and approved the submitted version.

## Funding

This work was funded by the National Institutes of Health (NIGMS-R35GM137957, F32GM139351) and the International Anesthesia Research Society (Frontiers in Anesthesia Research Award).

## Conflict of Interest

The authors declare that the research was conducted in the absence of any commercial or financial relationships that could be construed as a potential conflict of interest.

## Publisher’s Note

All claims expressed in this article are solely those of the authors and do not necessarily represent those of their affiliated organizations, or those of the publisher, the editors and the reviewers. Any product that may be evaluated in this article, or claim that may be made by its manufacturer, is not guaranteed or endorsed by the publisher.
